# Desktop-Stereolithography 3D Printing of a Polyporous Extracellular Matrix Bioink for Bone Defect Regeneration

**DOI:** 10.3389/fbioe.2020.589094

**Published:** 2020-11-06

**Authors:** Yunxiang Luo, Hao Pan, Jiuzhou Jiang, Chenchen Zhao, Jianfeng Zhang, Pengfei Chen, Xianfeng Lin, Shunwu Fan

**Affiliations:** ^1^Department of Orthopaedic Surgery, Sir Run Run Shaw Hospital, Medical College of Zhejiang University, Hangzhou, China; ^2^Key Laboratory of Musculoskeletal System Degeneration and Regeneration Translational Research of Zhejiang Province, Hangzhou, China; ^3^Department of Orthopaedic, The First Affiliated Hospital of Wenzhou Medical University, Wenzhou, China

**Keywords:** 3D printing, stereolithography, tendon extracellular matrix, polyethylene glycol diacrylate, calvarial defect

## Abstract

**Introduction:**

Decellularized tendon extracellular matrix (tECM) perfectly provides the natural environment and holds great potential for bone regeneration in Bone tissue engineering (BTE) area. However, its densifying fiber structure leads to reduced cell permeability. Our study aimed to combine tECM with polyethylene glycol diacrylate (PEGDA) to form a biological scaffold with appropriate porosity and strength using stereolithography (SLA) technology for bone defect repair.

**Methods:**

The tECM was produced and evaluated. Mesenchymal stem cell (MSC) was used to evaluate the biocompatibility of PEGDA/tECM bioink *in vitro*. Mineralization ability of the bioink was also evaluated *in vitro*. After preparing 3D printed polyporous PEGDA/tECM scaffolds (3D-pPES) via SLA, the calvarial defect generation capacity of 3D-pPES was assessed.

**Results:**

The tECM was obtained and the decellularized effect was confirmed. The tECM increased the swelling ratio and porosity of PEGDA bioink, both cellular proliferation and biomineralization *in vitro* of the bioink were significantly optimized. The 3D-pPES was fabricated. Compared to the control group, increased cell migration efficiency, up-regulation of osteogenic differentiation RNA level, and better calvarial defect repair in rat of the 3D-pPES group were observed.

**Conclusion:**

This study demonstrates that the 3D-pPES may be a promising strategy for bone defect treatment.

## Introduction

High-energy injuries or pathological fractures, such as tumors and inflammation, are the main reasons for bone defects, which create numerous challenges in the clinical setting and require bone grafting (Campana et al., 2014; Hwang et al., 2017; Zhang H. et al., 2019). As the gold standard for bone substitution in clinical surgeries, the autogenous bone grafts application is limited by the inadequate practicability, donor-site morbidity, and complicated surgical procedures (Sharif et al., 2016; Bez et al., 2018; Cabbad et al., 2019; Chen et al., 2019). Consequently, a more valid alternative procedure of BTE platform was proposed. BTE provides several benefits such as the rare spread of disease, lower infection or immunogenicity rate, various implantation materials, and wide availability (Hollister and Murphy, 2011). Many biomaterial substitutes have already been clinically applied due to their superior biological performance (Gibbs et al., 2016; Hassan et al., 2019).

Among all biological materials, the extracellular matrix (ECM) graft retains its natural structure and has high homology among different species, since it is derived from the biological tissue rather than the chemosynthesis materials, and it shows excellent effects in terms of the regulation of cellular adhesion, proliferation, migration, and differentiation (Pham et al., 2008). The tendon ECM (tECM) is rich in type-1 collagen, which can serve as a heterogeneous nucleation template to induce calcium and phosphorus (Ca-Pi) cluster formation (Xu et al., 2015; Thankam et al., 2018). In this biochemistry procedure, a self-assembled, pseudo-hexagonal array of collagen molecules participate and facilitate the Ca-Pi binding and nucleation (Xu et al., 2015). However, independent application of tECM to build a scaffold in BTE has several drawbacks; lack of sufficient mechanical support due to the original physical property, difficulty in forming a specific shape coinciding with the bone defect, and the lack of 3D micropore structure that is beneficial to the cell growth and differentiation (Narayanan et al., 2009).

To conquer the shortcomings that pure ECM is unable to provide, like adequate mechanical strength, the scaffold processing techniques are taken into consideration. Conventional scaffold processing techniques that fabricate various tissues, such as phase separation (Fang et al., 2019), freeze-drying (Grenier et al., 2019; Zhang L. et al., 2019), solvent casting (Ahn et al., 2018; Mao et al., 2018), gas foaming (Kaynak Bayrak et al., 2017; Catanzano et al., 2018), and electrospinning (Chan et al., 2019), cannot precisely control pore size, geometry, and interconnectivity of the scaffolds. However, 3D printing has emerged as a brand-new material processing approach, which largely overcomes these difficulties, allowing us to fabricate more bionic scaffolds for bone transplantation and to repair the bone defect in a clinical setting (Do et al., 2015). To ensure the 3D printing scaffold is built efficiently, hybridizing the natural ECM and synthetic polymer-based materials to create novel tissue-engineered scaffolds seems feasible because, in this method, the advantages, including the biocompatibility of the ECM, and the superior physical properties of the synthetic polymer-based materials, are both fully embodied (Lee et al., 2014). Though traditional 3D printing provides lots of benefits, creating a complicated 3D scaffold with natural ingredients of biological origin, such as fibrin, gelatin or hyaluronic acid, seems impractical (Knowlton et al., 2016). Stereolithography (SLA), a simple, user-friendly photo-crosslinked biomaterial printing with high resolution, gives us a brand-new solution to achieve bone regeneration (Do et al., 2015; Knowlton et al., 2016).

**SCHEME 1:**
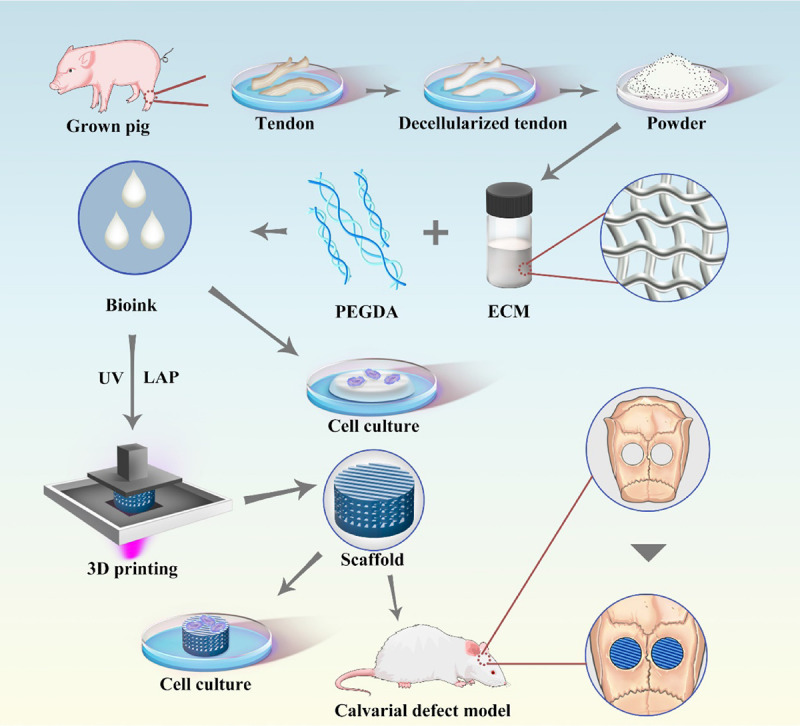
Schematic illustration of the polyporous tECM bioink 3D printing scheme, included PEGDA/tECM bioink preparation, tECM hydrogel for cell culture *in vitro* and SLA-based PEGDA/tECM bioprinting that applied to calvarial defect implantation.

Here, we focus on the 3D printing-based scaffolds using the hybridization of tECM and synthetic polymer-based materials to fabricate a highly interconnected architecture bone grafts scaffold (Scheme 1). This 3D printed polyporous PEGDA/tECM scaffolds (3D-pPES) could promote the mesenchymal stem cell (MSCs) proliferation and migration in the defect regions. The promising bone defect regeneration could be achieved using this novel repair system.

## Materials and Methods

All animal experiments *in vivo* were conducted following the principles and procedures of the National Institutes of Health (NIH) Guide for the Care and Use of Laboratory Animals and the guidelines for animal treatment of Sir Run Shaw Affiliated Hospital of Zhejiang University School of Medicine (Hangzhou, Zhejiang).

### Preparation of the tECM

First, grown pigs were purchased at a local slaughterhouse in Hangzhou, and ten porcine tendons were harvested from their hind legs. The tendons were exposed to three continuous freeze-thaw (−80–37°C) cycles. Then, the tissues were immersed in 1% Triton X-100 solution for 24 h, 1% sodium dodecyl sulfate for 3 h and 200 U mL^–1^ DNAase at 37°C for 12 h.

Next, 4′, 6-diamidino-2-phenylindole (DAPI), hematoxylin and eosin (H&E), Masson’s trichrome staining and a DNA assay were performed to evaluate the decellularized efficiency. Using a Universal Genomic DNA Kit (CW Biotech, Beijing, China) and a microplate spectrophotometer (260 nm, Thermo Fisher Scientific, Waltham, MA, United States), DNA contents were measured. Meanwhile, a hydroxyproline (Hyp) assay kit (Nanjing Keygen Biotech Co., Ltd., Nanjing, China) was used to measure the collagen content. The decellularized tECM was dehydrated, ground for digestion, and adjusted to a neutral pH (7.4).

### Physical Performance Measurement of the Hydrogels

The physical properties of bioinks with different compositions were determined. Exactly 10% (w/v) polyethylene glycol diacrylate (PEGDA) and 0.25% (w/v) lithium acylphosphinate photo-initiator (LAP) were added to the tECM hydrogel obtained previously, as described above, then the PEGDA/tECM pre-gel was crosslinked for 15 s at 375 nm of UV light exposure. Isostatic compression tests of the hydrogels were conducted in a dry state at 25°C using a universal testing system (Instron 5567, United States). The weight swelling ratio, Q, was calculated using the following equation:

$$Q=\frac{\mathrm{Swelling}\,\mathrm{mass}\,\left(\mathrm{Ws}\right)}{\mathrm{dry}\,\mathrm{mass}\,\left(\mathrm{Wd}\right)}\times 100\%$$

The coagulation time of several types of hydrogels with different tECM concentrations was tested on the print plane. The 3D microstructure of the lyophilized PEGDA/tECM hydrogels was observed using scanning electron microscopy (SEM). An Icon atomic force microscope (Dimension Icon, Bruker, Billerica, MA, United States) was used to observe the PEGDA hydrogel and scaffold.

### Cell Viability and Metabolic Activity Assays of the Hydrogels

For all the experiments on cell viability and metabolic activity assays, C57BL/6 bone marrow-derived MSCs were used, which were purchased from Cyagen Biosciences (MUBMX-90011, Santa Clara, United States). Live-Dead Cell Staining Kit (Thermo Fisher Scientific, Waltham, MA, United States) was used to assay the MSCs viability and CCK-8 kit (Cell Counting Kit-8; Dojindo Laboratories, Kumamoto, Japan) was used to test the metabolic activity of MSCs growing on the hydrogels. Using the same experimental conditions, we also carried out the cytotoxicity test of LAP.

### Spontaneous Biomineralization of the Hydrogels *In vitro*

The hydrogel specimens (pure PEGDA group and PEGDA/tECM group) were produced and merged into the modified simulated body fluid (m-SBF; 1.67 × 10^–3^ M CaCl_2_, 9.5 × 10^–3^ M Na_2_HPO4, 150 × 10^–3^ M NaCl, and 100 μg/mL^–1^ polyaspartic acid) for 2 and 4 weeks. Micro-CT scanning (Siemens Inveon, Eschborn, Germany) was used following a scanning protocol of 80 kV, 500 mA, and 14.97 mm isotropic resolution. Data were obtained and analyzed using Inveon Research Workplace v. 2.2 software (Siemens, Munich, Germany).

### Biofabrication of 3D-pPES Using Dynamic Projection SLA

A digital light processing (DLP) chip (Discovery 4000; Texas Instruments, Dallas, TX, United States), a replaceable UV light source at 375 nm wavelength (OmniCure S2000; EXFO, Quebec City, QC, Canada), and XYZ stages made up our SLA printing system. Pre-designed user-defined computer-aided-design (CAD) files could be read and converted into printable programs by the DLP chip. The light from the UV light source was directed onto the print plane of the bioink through an optically-specific lens (Edmunds Optics, Barrington, NJ, United States). Catalyzed by LAP, the bioink reacted rapidly in the projected space and quickly solidified into a 3D scaffold at certain physical strength.

### Cell Migration and Cytotoxicity Assay of 3D-pPES

CCK-8 kit (Cell Counting Kit-8; Dojindo Laboratories, Kumamoto, Japan) was used to test the metabolic activity of MSCs growing in the 3D-pPES lixivium. We conducted control studies using static cultures of MSCs in Transwell plates (Corning Inc., Lowell, MA, United States), containing porous polyester membrane inserts (0.33 cm^2^, 0.4 μm pores), to detect cell migration ability.

### Evaluation of the Osteoinductive Activity of the 3D-pPES *In vitro*

The scaffolds, containing 1% (w/v) tECM, were placed in 96-well plates, at 1 × 10^5^ cells/well MSCs. Both DMEM supplemented with 10% FBS and 100 μg/mL streptomycin were added. Osteoblastic induction medium (Sigma Aldrich Corp., St. Louis, MO, United States) was added to incubate MSCs. On day 7, real-time quantitative polymerase chain reaction (RT-qPCR) was performed to measure the expression of *ALP, Runx2, Col1α1, OCN*, and *OPN. GAPDH* was referred to as a quantitative control for RNA levels. The primer sequences are listed in Supplementary Table S1.

### Protein Mass Spectrometry of the tECM

Deformation and reduction of the protein sample were performed for proteomic experiments. Protein concentration was determined using BCA assay. Peptide samples were analyzed using nano-LC-MS/MS. Sequences were mapped based on gene ontology (GO) terms^1^ to determine the biological and functional properties of all identified proteins. Meanwhile, we employed hypergeometric tests to perform GO enrichment analysis.

### Repair Assay in the Rat Critical-Sized Calvarial Defect Model

Twelve one-month-old Sprague Dawley rats were purchased and raised individually in cages. After anesthesia, bilateral full-thickness critical-sized calvarial defects (4 mm in diameter) were created. Details of each group of operation are shown in Supplementary Table S2. The skulls were collected and embalmed in 4% paraformaldehyde. Repair assay in the rat critical-sized calvarial defect model was implemented using micro-CT scanning. The specimens were cut along the coronal plane for H&E, Masson’s trichrome, and Goldner trichrome staining.

### Statistical Analysis

Data are presented as mean ± standard deviation (SD) of at least three experiments with similar results. Experiments were run in triplicate unless stated otherwise. Either one−way ANOVA or Student’s *t*-test was applied to assess the differences between the means. ^∗^*P* < 0.05, ^∗∗^*P* < 0.01, ^#^*P* < 0.05, and ^##^*P* < 0.01 were considered statistically significant.

## Results

### Preparation of a Decellularized Tendon With Potential Biological Functions

**FIGURE 1:**
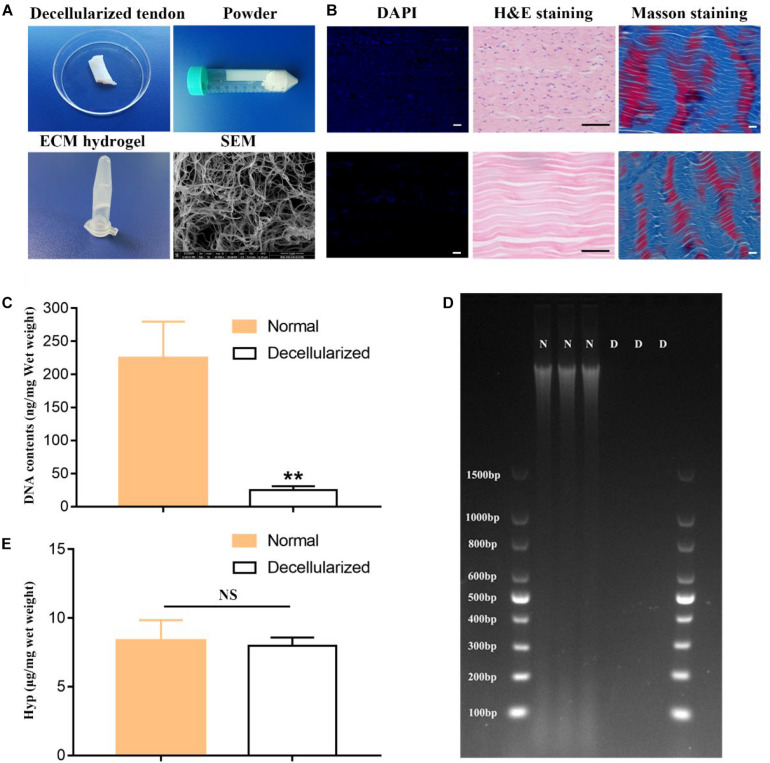
Fabrication and characterization of the hydrogel derived from decellularized tendon. **(A)** General and SEM Images of tendon decellularization and preparation of hydrogel. **(B)** H&E and DAPI staining confirmed cell removal; Masson trichrome staining confirmed collagen retention. Scale bar = 10 μm. Quantitative detection of DNA removal retention **(C,D)** and collagen **(E)**. **P < 0.01 compares to normal group.

The microstructure, composition and biological functions of the decellularized tendon were carefully analyzed. After decellularization, the tendon pieces were ground into powder, which was dissolved in acid to form a gel (Figure 1A). The tECM mainly presented a filamentous structure observed by SEM (Figure 1A). DAPI and H&E staining (Figure 1B) showed the absence of the nucleus. Well-organized fibrous structures were observed via HE staining (Figure 1B). Masson trichrome staining confirmed excellent collagen retention as well (Figure 1B). The DNA content decreased approximately by 97% (Figures 1C,D) after the decellularization. There was little change in collagen content before and after decellularization (Figure 1E).

### Physical Properties of the Hydrogels

**FIGURE 2:**
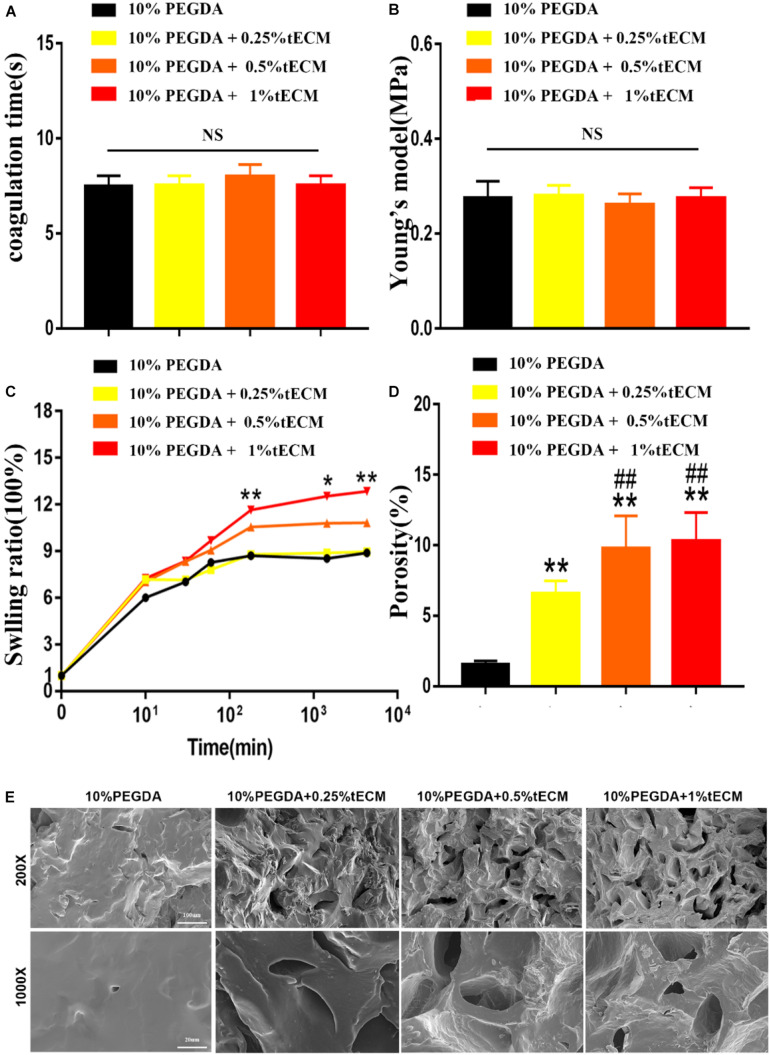
Physical properties of the bioink and hydrogel: coagulation time **(A)**, Young’s modulus **(B)**, swelling ratio **(C)** and porosity **(D,E)**. The hydrogel was observed via SEM **(E)**. **P < 0.01 compares to 10% PEGDA group. ^##^*P* < 0.01 compares to 10% PEGDA + 0.25% tECM group. *P < 0.05 compares to 10% PEGDA group.

The physical properties of the bioink and hydrogels were further observed from several aspects. Both coagulation time and Young’s modulus showed no visible difference when the concentration of the tECM changed from 0 to 1% (Figures 2A,B). The representative curve of elastic force generated by the pressure with the displacement of compression deformation is shown in Supplementary Figure S1. Rapid swelling and water absorption were observed within the first 100 min, then the swelling ratio verged to extremely slow after 3 h (Figure 2C). Moreover, 1% tECM greatly increased the swelling ratio of the PEGDA hydrogels. As the concentration of the tECM increased, an increasing number (10%) of holes appeared, observed via SEM, which contributed to the increase of the hydrogel porosity (Figures 2D,E). We assumed that tECM hydrogel greatly improved the physical characteristics of the hydrogel. The data above indicated that the PEGDA/tECM hydrogel was endowed with good hygroscopicity and appropriate mechanical properties.

### PEGDA/tECM Hydrogels Promote Cellular Proliferation and Biomineralization *In vitro*

**FIGURE 3:**
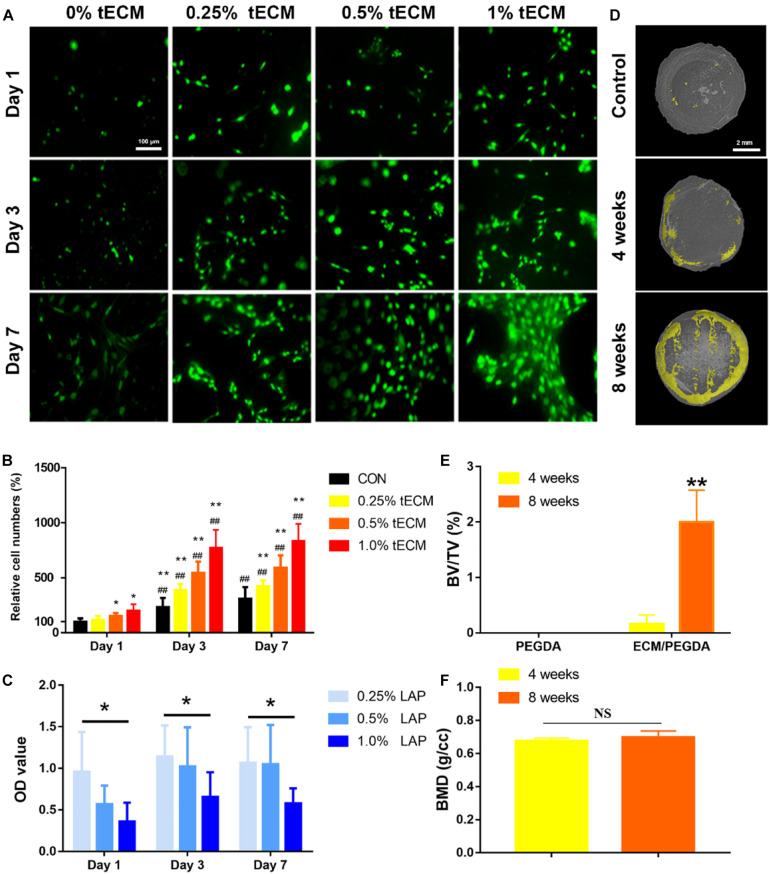
Evaluation of Increased cellular proliferation and biomineralization *in vitro* by the PEGDA/tECM hydrogel. **(A)** Cell proliferation on the PEGDA, 0.25% tECM/ PEGDA, 0.5% tECM/ PEGDA, 1% tECM/ PEGDA hydrogels. **(B)** Quantitative analysis of Cell proliferation on the PEGDA, 0.25% tECM/ PEGDA, 0.5% tECM/ PEGDA, 1% tECM/ PEGDA scaffolds. **(C)** Quantitative analysis of Cell proliferation at different concentrations of LAP. **(D)** Representative Micro-CT scans images of PEGDA hydrogels for 8 weeks and PEGDA/tECM hydrogels for 4 and 8 weeks. Quantitative analysis of the mineralization presented in using BV/TV **(E)** and BMD **(F)** values. *P < 0.05, **P < 0.01 compares to control group at the same time. ^##^*P* < 0.01 compares to the same group on day 1. BV/TV: trabecular bone volume fraction, BMD: Bone mineral density. Scale bars are shown in the figure.

The cell viability assay showed that the cell proliferation rate was significantly higher on the PEGDA/tECM hydrogel compared to the control hydrogel (Figures 3A,B). All groups containing tECM showed a significant difference compared to the control group; this difference was observed from day 1. Moreover, as the concentration of the tECM increased, the proliferative capacity of the cell improved (Figures 3A,B). To create a scaffold with a suitable cytocompatibility, we tested the cytotoxicity of LAP (Figure 3C). The quantitative analysis proved that high concentration (1% wt) of LAP showed more potent cytotoxicity, while the bioink, containing low concentration (0.25% wt) of LAP, met the UV crosslinking requirements of 3D printing and showed better biocompatibility (Figure 3C). In our following experiment, the bioink that contained low concentration (0.25% wt) of LAP and high concentration of tECM (1% wt) was used uniformly. Then the PEGDA hydrogels and PEGDA/tECM hydrogels were immersed in m-SBF. Micro-CT results of the PEGDA/tECM group showed poor mineralization and relatively low BV/TV at 4 weeks, while the results at 8 weeks increased greatly (*P* < 0.05, Figures 3D–F). However, little change was observed in the PEGDA group due to the absence of tECM (Figures 3D,E). Hence, PEGDA/tECM hydrogels were better than PEGDA hydrogels at coordinating Ca-Pi deposition.

### 3D Printing of Polyporous PEGDA/tECM Bioink

**FIGURE 4:**
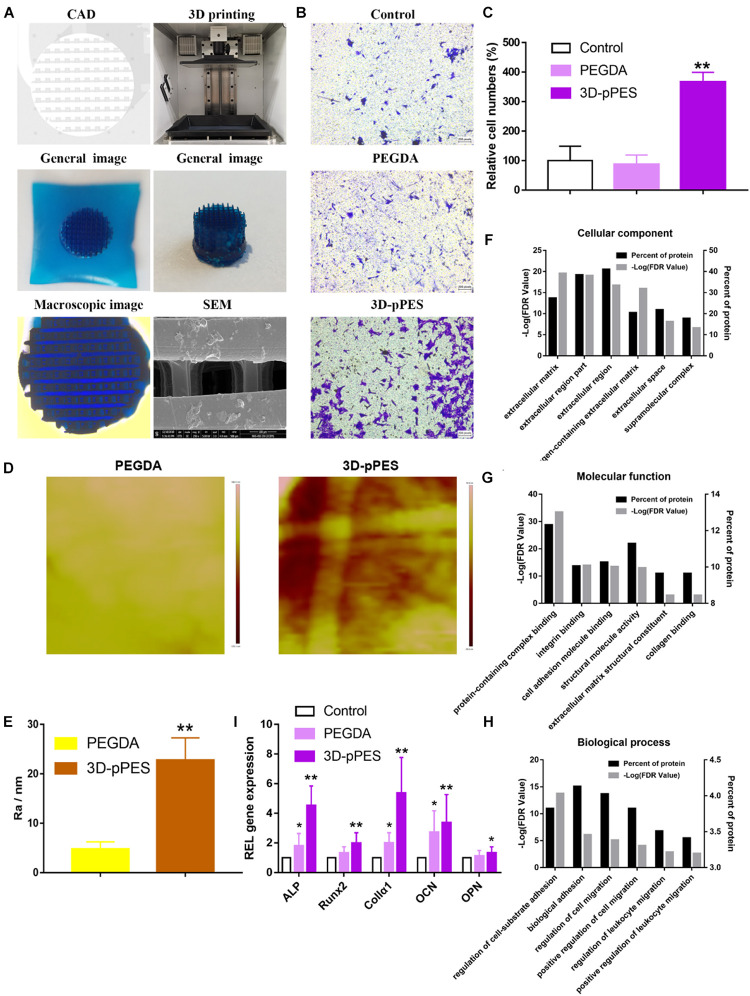
3D printing of polyporous tECM bioink and scaffold promoting migration and osteogenic differentiation. **(A)** CAD file, printing process, and scaffold (stained with methylene blue dye) printed by the 3D printer. The 3D-pPES was observed under microscopy and SEM. **(B)** Transwell migration assay of different treatments. Scale bars = 100 μm. **(C)** Quantitative analysis of migrating cells. **(D)** Roughness image by AFM within the scope of 1 μm^2^. **(E)** Quantitative analysis of roughness value Ra (*n* = 5). **(F–H)** GO classification of tECM proteins. Scale bars are shown in the figure. **(I)** qPCR quantification of the relative mRNA expression of Runx2, ALP, OCN, OPN, and Col1α1 in osteoblasts cultured for 14 days on the hydrogels (*n* = 6). *P < 0.05 compares to control group. **P < 0.01 compares to control group.

Computer-aided-design files (Figure 4A) were used to produce the virtual mask and transmit UV light to the PEGDA/tECM bioink to print polyporous scaffolds. The structural formula of PEGDA and LAP are shown in Supplementary Figure S2. The line chart of the real-time temperature of the bioink in the printing process showed that the temperature was kept below 37°C (Supplementary Figure S3). The SLA-based technique successfully yielded a polyporous scaffold with abundant and dense pipelines that could be observed both by the naked eye and under a microscope (Figure 4A). These channels were also visible by SEM after lyophilizing the scaffold for 24 h (Figure 4A).

### Increased Migration and Osteogenic Differentiation Triggered by the 3D-pPES

Before the MSCs were reseeded for cell differentiation, non-cytotoxicity of the 3D-pPES was confirmed (Supplementary Figure S4). Quantitative analysis of cell numbers showed that cell migration increased 3.6 times in the 3D-pPES group compared to the control group and PEGDA group (Figures 4B,C). Roughness image showed 3D-pPES group has a rougher surface than PEGDA group (Figures 4D,E). It confirmed that the addition of ECM made the surface of the scaffold rougher. Proteomic analysis of tECM was also performed to divide the identified proteins into three classes (Figures 4F–H). GO cellular component and molecular function analysis confirmed the successful retention of various ECM structural constituents after decellularization (Figures 4F,G), which provided the bionic environment for cell growth. GO biological process analysis showed that the tECM contained proteins that function as regulation of cell adhesion and migration (Figure 4H), which might have contributed to the migration in Figures 4B,C. The result of the proteomic analysis corresponded with that of the Transwell assay. Osteogenic differentiation experiment *in vitro* showed conformably upregulated expression of genes associated with osteogenesis (Runx2, ALP, Col1α1, OCN, and OPN) after 14 days of induction (Figure 4I). The rougher 3D-pPES has a more pronounced effect on osteogenic differentiation than PEGDA. These findings indicated that the 3D-pPES had a positive effect on cell migration and osteogenic differentiation.

### Bone Regeneration Enhancement Triggered by the 3D-pPES

**FIGURE 5:**
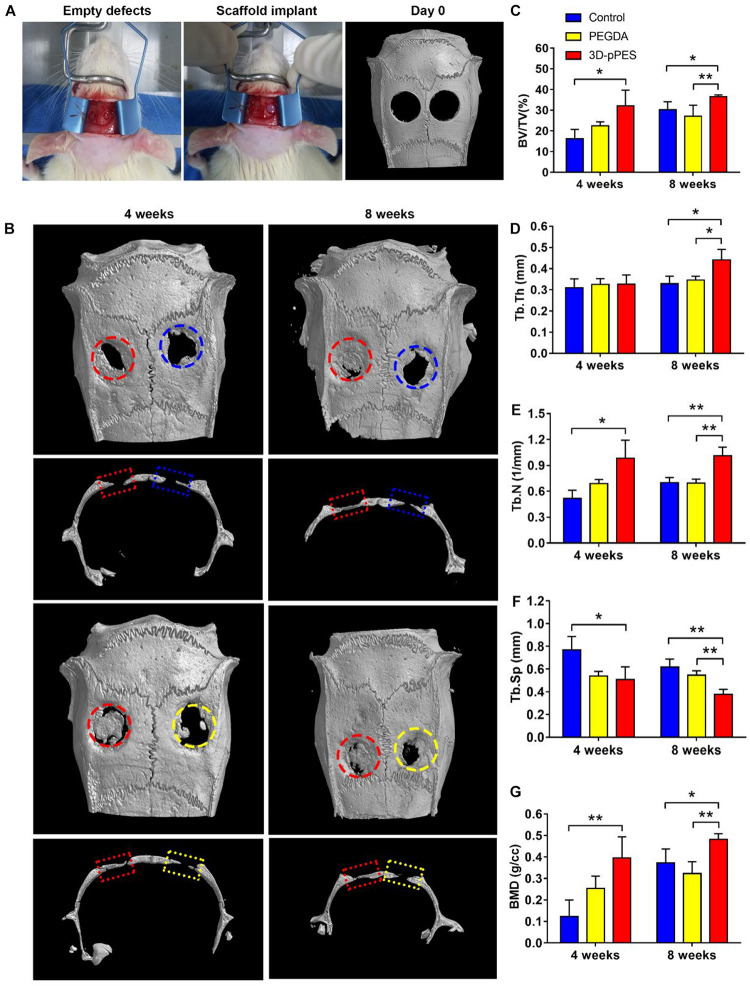
Bone regeneration in the calvarial bone defect model. **(A)** Surgical procedure and micro-CT scanning of the calvarial defect after surgery on day 0. **(B)** Mineralization of the calvarial defect was evaluated by micro-CT and bone defect healing at 4 and 8 weeks after gel implantation was showed. **(C–G)** Quantitative analysis of **(C)** BV/TV, **(D)** Tb.Th, **(E)** Tb.N, **(F)** Tb.Sp, and **(G)** BMD new bone formation area of the regenerated bone 4 and 8 weeks after implantation. BV, bone volume; TV, total volume; Tb.Th, Trabecular thickness; Tb.N, trabecular number; Tb.Sp, Trabecular separation; BMD, bone mineral density. The blue circle/rectangle represented the original defect of control group, the yellow circle/rectangle represented the original defect of PEGDA group, the red circle/rectangle represented the original defect of 3D-pPES group.

The relative efficacy of PEGDA and PEGDA/tECM hydrogels in promoting new bone formation was evaluated in rats with induced critical-sized calvarial bone defects (Figure 5A) at 4 and 8 weeks after surgery. Observation and analysis of the regenerated bone were successfully conducted, applying micro-CT scanning. Newly-formed bone was observed in all groups. However, the maximum amount of mineralized bone was measured in the PEGDA/tECM group (Figure 5B), along with the maximum value in BV/TV, Tb.Th, Tb.N, and BMD (Figures 5C–G). In contrast, Tb.Sp value of the PEGDA/tECM group was the lowest since it was inversely proportional to the BMD value (Figure 5F). Notably, the increased value in BV/TV, Tb.Th, Tb.N, and BMD of the PEGDA group was observed. A similar conclusion could be drawn that a rigid surface of PEGDA without tECM was slightly effective in osteogenic differentiation and biomineralization.

**FIGURE 6:**
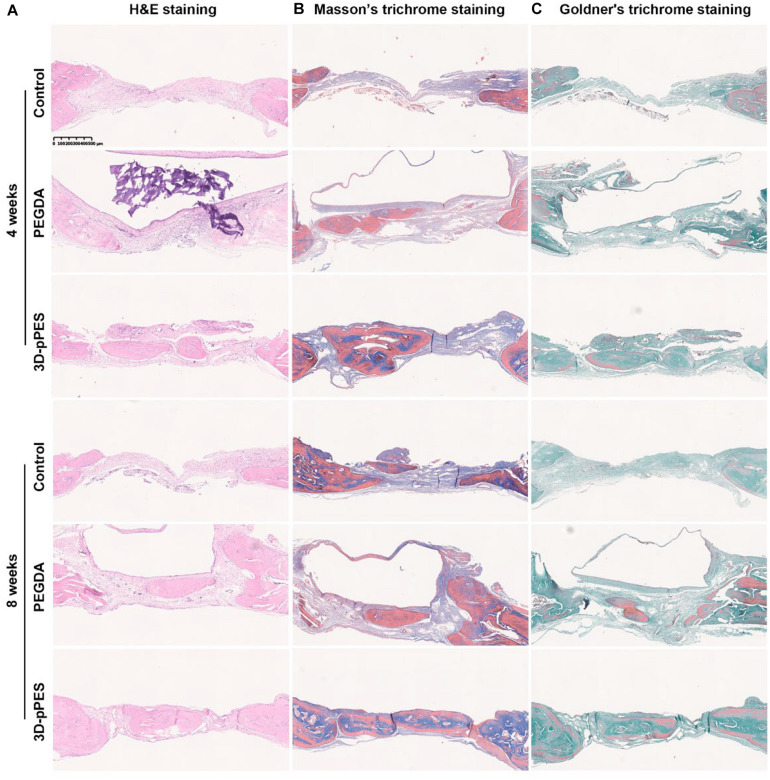
Osteogenesis after hydrogel implantation showed by histological staining. **(A)** H&E staining of the calvarial defects at 4 and 8 weeks after hydrogel implantation. **(B)** Masson’s trichrome staining of the calvarial defects at 4 and 8 weeks after hydrogel implantation, new bones were shown in dark blue. **(C)** Goldner’s staining of the regenerated bone in calvarial defects, fibroblastic connective tissue was shown in light green, immature woven bone and osteoid tissue were shown in red. mature bone island (lamellar bone) was showed in dark green. Scale bar = 500 μm.

From the H&E stained images (Figure 6A), it was clearly seen that the bone defects of the control groups and PEGDA groups were partly recovered, with the bone defect still unjoined. Little evidence supporting the new bone formation was found in the control group after 4 weeks. Denser new bone and more bone-like tissues near the border of the bone defect were seen in the PEGDA/tECM group. Markedly, the amount of the new bones observed in the PEGDA/tECM group were high compared to the other two groups (Figure 6B). Goldner’s trichrome staining (Figure 6C) revealed that in PEGDA and PEGDA/tECM groups, immature woven bone and osteoid were formed in the defects, while the formation of mature lamellar bone, and even new bone marrow by the end of the observation period, were observed in the PEGDA/tECM group. All the evidence above showed a reasonable inference that the 3D-pPES had the best therapeutic effect in bone regeneration in the rat model. Meanwhile, degradation experiments *in vitro* (Supplementary Figure S5) further proved that the optimized degradation performance may further promote the function of bone repair.

## Discussion

Summarizing the experimental results above, our article is the first to report this innovative composite scaffold fabricated by SLA to repair bone defect that had advantage of both the biocompatibility of ECM and mechanical strength of traditional synthetic materials. The innovative scaffold was rich in various bioactive factors, had micropore structure on the surface and interconnectivity of macrostructures, attracted cells, and promoted osteogenic differentiation. Ultimately, experimental results showed that both *in vitro* and *in vivo* experiments resulted in apparent facilitation in terms of osteogenesis.

The ECM has been reported commonly in the field of bone and other parts repair (Pham et al., 2008; Pacelli et al., 2017; Matai et al., 2020). Our team has published relevant literature before. For instance, (Qiu et al., 2020) has fabricated an injectable periosteal ECM hydrogel that dynamically integrates multiple biological functions, promotes angiogenesis and osteogenesis at the defect site. The whole dynamic process contains bone formation, remodeling and repair, which involves cell migration, ECM assembly, osteocyte embedding, and bone resorption (Shiflett et al., 2019). Our experimental results have proved the effect on cell growth, migration, and differentiation. However, the physical properties and spatial structure of this original material need to be improved (Narayanan et al., 2009).

In this study, the physical and mechanical strength of the material was significantly improved by adding PEGDA. As reported previously (Engler et al., 2006), naive MSCs differentiated toward a specific lineage and committed to phenotypes with extreme sensitivity to tissue-level elasticity, and when tissue-level elasticity of the matrices mimicked a collagenous bone, the outcome was osteogenic (Engler et al., 2006; Caliari et al., 2016). PEGDA (∼0.3 MPa) improved the hardness of the material and promoted osteogenic differentiation of MSCs. In fact, in our animal experiments, there were also some weak positive results of osteogenesis in the PEGDA group.

In contrast, the addition of tECM improved the biocompatibility of traditional synthetic materials. Various parameters, including surface properties, mobility and solute diffusion, were affected by the swelling ratio, an essential material parameter in tissue engineering (Stephanopoulos et al., 2013). Microscopically, both the pore size of the polymer and the interaction between the solvent and polymer affect the value of swelling ratio (Du et al., 2008). The addition of tECM greatly improved the swelling degree of the material, and as the concentration of tECM increased, the swelling degree also increased, which was closely related to the hydrophilicity of tECM, as it contains numerous hydrophilic components. The tECM component increased the porosity of the hydrogel. The scaffold with more pores and a larger pore size creates favorable conditions for cell survival, adhesion, and migration (O’Brien et al., 2005, 2007).

To further improve the external form and internal permeability of the PEGDA/tECM hydrogel, SLA technology was applied in the fabrication of this novel scaffold. To date, A challenge that inkjet bioprinting, a traditional printing method, faces is that it is challenging to print vertical 3D structures, while SLA avoids this problem entirely (Wang et al., 2015; Bhattacharjee et al., 2018). Other drawbacks inkjet printing has include shear stress that damage cells, and print nozzle blockage (Wang et al., 2018). In contrast, the SLA printing speed is not affected by plane complexity, while the number of printing layers determines the printing time (Wang et al., 2015). Also, each printing detail may extend the printing time in traditional inkjet bioprinting. Consequently, complex 3D shapes are more likely to be created by SLA without extending the printing time (Wang et al., 2015). The printing time of a customized scaffold is extremely important since urgent surgical treatment is crucial in accidental trauma (Rauch et al., 2019).

Compared with traditional synthetic materials, 3D-pPES has been confirmed to have a rougher surface. Micro-roughness can affect the type of integrins produced by cells, promoting those subunits associated with bone proteins, such as α_2_ and β_1_ (Olivares-Navarrete et al., 2008). Moreover, micro-rough surface evoked accelerated gene expression of the bone matrix molecules osteopontin and osteonectin and up-regulated of bone sialoprotein, collagen III and integrins (Salvi et al., 2015). In fact, both 3D micropore architectural structure and micro-rough surface of 3D-pPES accelerate the MSCs osteogenic differentiation.

Polyethylene glycol diacrylate/tECM hydrogel showed better capability in coordinating Ca-Pi deposition, which led to high biomineralization. The cell osteogenesis induction experiment *in vitro* also proved that PEGDA/tECM hydrogels significantly and conformably upregulated the expression of genes associated with osteogenesis. There is significant evidence that endochondral (EC) ossification occurs in ECM grafts (Dennis et al., 2015) and our results agree with this conclusion. The osteogenic properties of our 3D-pPES were proved by *in vivo* experiments. The repair of critical-sized calvarial bone defects in the 3D-pPES group showed the highest quality and the most substantial quantity of new mineralized bone formation. Meanwhile, from the results of tissue slices from animal experiments, islands of new bone formation were observed in the 3D-pPES group. Moreover, denser new bone and more bone-like tissues near the border of the bone defect were observed in the 3D-pPES group.

Degradation experiment *in vitro* proved that the 3D-pPES have the advantage of faster degradation. First, ECM has multiple biological enzymes to accelerate the degradation of scaffold (Pham et al., 2008). Second, the 3D-pPES greatly increased the surface area of the material. Moreover, it has been reported that scaffold microarchitecture profoundly influences macrophage adhesion, infiltration and differentiation (Kurpinski et al., 2010). Once invading into the scaffold microarchitecture, macrophages secrete reactive oxygen species (ROS) and hydrolytic enzymes, contributing to oxidative and enzymatic biomaterial degradation, respectively (Wissing et al., 2019). Meanwhile, the scaffolds with larger pore size on the micron scale can promote the oxidative degradation of the synthetic scaffolds (Versaevel et al., 2012). Optimized by multiple factors, 3D-pPES showed superior degradation and repair performance.

We can reasonably speculate that a relatively biomimetic bone repair process has been achieved via intramembranous ossification (Dennis et al., 2015). MSCs proliferate intensively and differentiate into osteoblasts, forming ossification center. The ossification center acts as a primed template for subsequent osteoblast infiltration, woven bone ossification, and bone remodeling restore healthy lamellar bone architecture (Dennis et al., 2015; Serra-Vinardell et al., 2020). Further work is needed to confirm the inference of these molecular mechanisms.

## Conclusion

In this study, we revealed that PEGDA/tECM hydrogel is a greatly enriched small aperture with a traditional synthetic material surface. It overcomes the drawbacks of insufficient mechanical strength of the traditional ECM and promotes cell growth. Using SLA, our 3D-pPES promote cell migration, initiate MSCs differentiation toward osteogenesis. In conclusion, promising repair efficacy of the 3D-pPES is confirmed in a bone defect model.

## Data Availability Statement

The original contributions presented in the study are included in the article/Supplementary Material, further inquiries can be directed to the corresponding authors.

## Ethics Statement

The animal study was reviewed and approved by Sir Run Shaw Affiliated Hospital of Zhejiang University School of Medicine.

## Author Contributions

All authors listed have made a substantial, direct and intellectual contribution to the work, and approved it for publication.

## Conflict of Interest

The authors declare that the research was conducted in the absence of any commercial or financial relationships that could be construed as a potential conflict of interest.

## Footnotes

1 http://geneontology.org/
