# Supplementary material for: Desktop-Stereolithography 3D Printing of a Polyporous Extracellular Matrix Bioink for Bone Defect Regeneration
Source: Front Bioeng Biotechnol. 2020 Nov 6;8:589094. doi: 10.3389/fbioe.2020.589094 (PMC7677189; doi:10.3389/fbioe.2020.589094)
Supplement: Supplementary file 1 [file Data_Sheet_1.docx]

**Table S1**

| Table S1. Primers used in qPCR | |
| --- | --- |
| Primer | Sequence |
| Col1α1 forward | CCCTGGTCCCTCTGGAAATG |
| Col1α1 reverse | GGACCTTTGCCCCCTTCTTT |
| Runx2 forward | ATACCCCCTCGCTCTCTGTT |
| Runx2 reverse | ACATAGGTCCCCATCTGCCT |
| ALP forward | TTCATAAGCAGGCGGGGGA |
| ALP reverse | GGTGTACCCTGAGATTCGTCC |
| OCN forward | CCCAGACCTAGCAGACACCA |
| OCN reverse | GCCGGAGTCTGTTCACTACC |
| OPN forward | TGGAGAGGTAGAAAAGGCACA |
| OPN reverse | CAAACACACTCTTGGCACCAC |
| GAPDH forward | ACCCAGAAGACTGTGGATGG |
| GAPDH reverse | CACATTGGGGGTAGGAACAC |

**Table S2**

Table S2. Different operation procedure of Each group

| Group | calvarial defects | | Harvesting time |
| --- | --- | --- | --- |
|  | Left | Right |  |
| I | Blank | 3D-pPES  3D-pPES  3D-pPES  3D-pPES | 4 weeks |
| II | PEGDA hydrogel |  | 4 weeks |
| III | Blank |  | 8 weeks |
| IV | PEGDA hydrogel |  | 8 weeks |

**Supplemental Fig. S1**


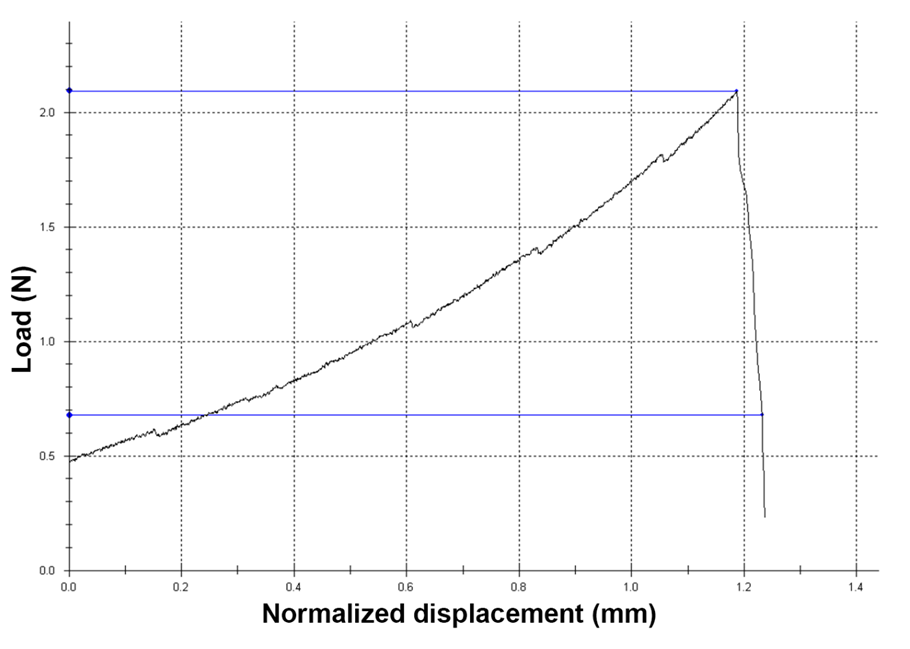


Supplemental Fig. S1 Representative stress–strain curve of the 3D-pPES. The initial linear portion of the stress–strain data showed the process of elastic deformation of a hydrogel. Room temperature: 25℃, Relative humidity: 50%.

**Supplemental Fig. S2**

**
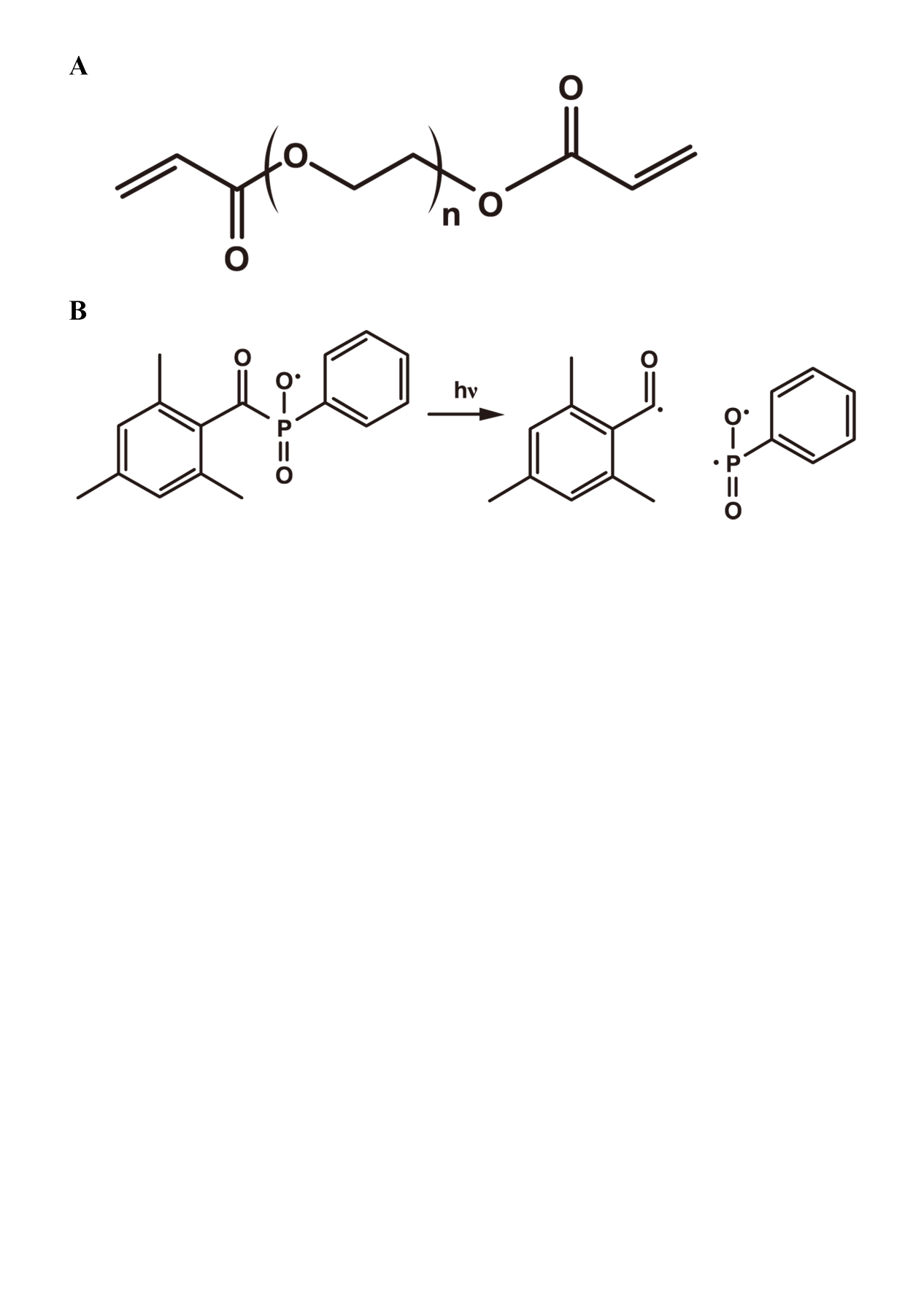
**

Supplemental Fig. S2 The structural formula of PEGDA (A) and LAP (B).

**Supplemental Fig. S3**


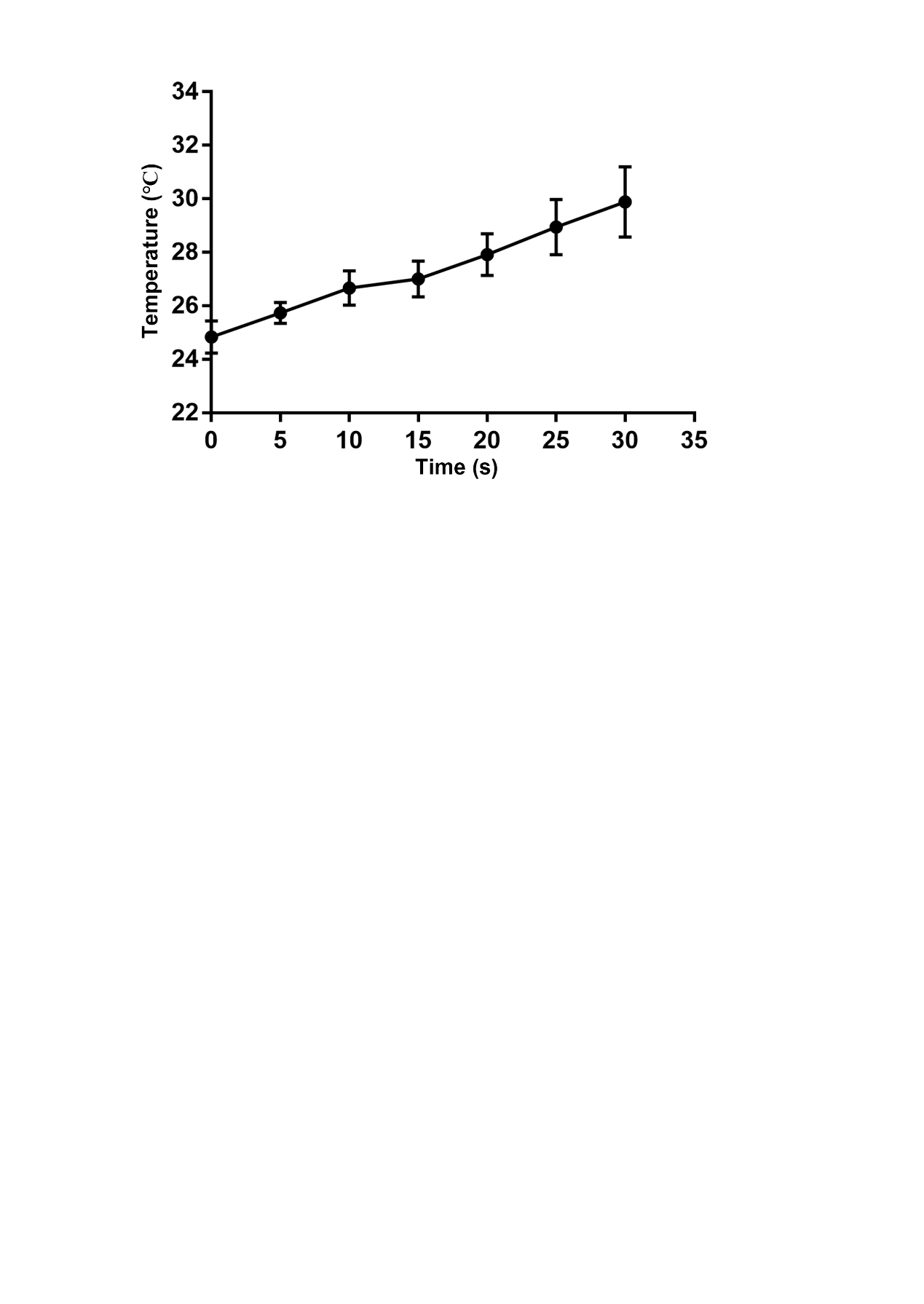


Supplemental Fig. S3 Time-temperature curve of the entire printing process. Room temperature: 25℃.

**Supplemental Fig. S4**


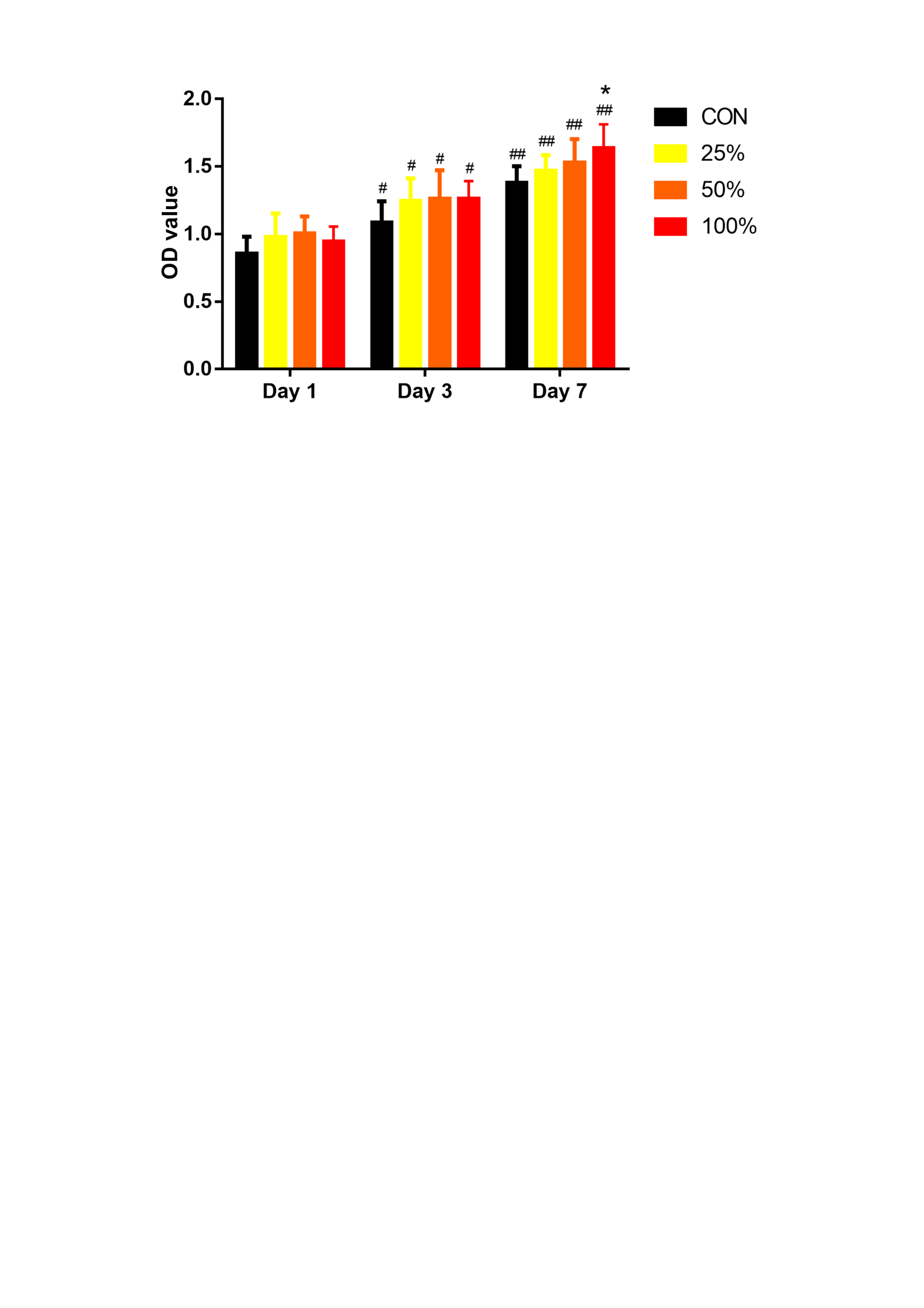


Supplemental Fig. S4 Qualitative analysis of the 3D-pPES leaching solution Cytotoxicity. *P < 0.05, compares to control group at the same time. #P < 0.05, ##P < 0.01 compares to the same group on day 1.

**Supplemental Fig. S5**


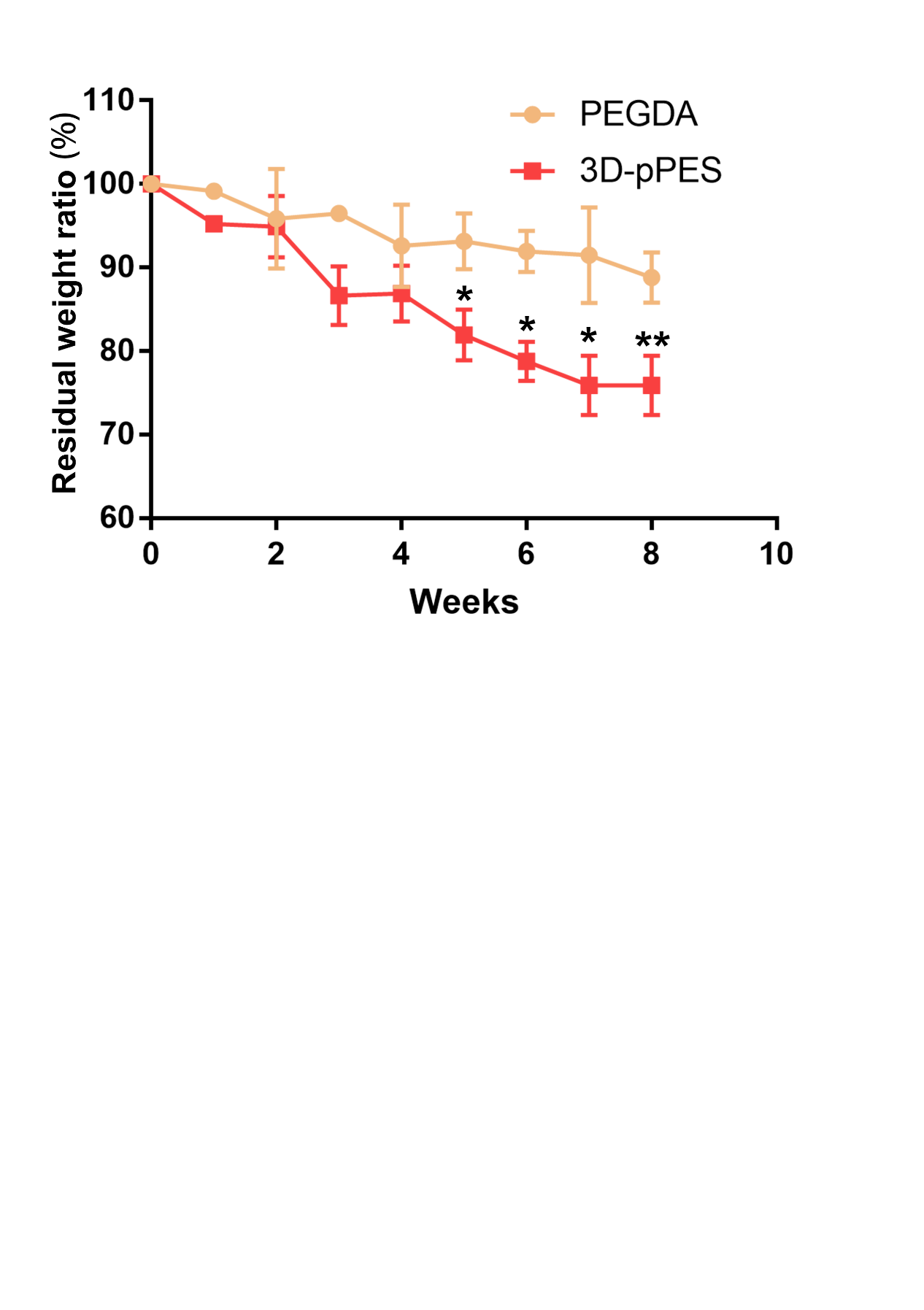


Supplemental Fig. S5 The degradation curve of the PEGDA and 3D-pPES *in vitro*. *P < 0.05. **P < 0.01.
